# SARS-CoV-2 Induces Cytokine Responses in Human Basophils

**DOI:** 10.3389/fimmu.2022.838448

**Published:** 2022-02-24

**Authors:** Srinivasa Reddy Bonam, Camille Chauvin, Laurine Levillayer, Mano Joseph Mathew, Anavaj Sakuntabhai, Jagadeesh Bayry

**Affiliations:** ^1^ Institut National de la Santé et de la Recherche Médicale, Centre de Recherche des Cordeliers, Sorbonne Université, Université de Paris, Paris, France; ^2^ Functional Genetics of Infectious Diseases Unit, Department of Global Health, Institut Pasteur, Paris, France; ^3^ EFREI, Villejuif, France; ^4^ Centre National de la Recherche Scientifique (CNRS), UMR2000, Paris, France; ^5^ Department of Biological Sciences & Engineering, Indian Institute of Technology Palakkad, Palakkad, India

**Keywords:** basophils, SARS-CoV-2, COVID-19, IL-3, Caco-2 cells, IL-13, IL-4, epithelial cells

## Abstract

Basophils play a key role in the orientation of immune responses. Though the interaction of SARS-CoV-2 with various immune cells has been relatively well studied, the response of basophils to this pandemic virus is not characterized yet. In this study, we report that SARS-CoV-2 induces cytokine responses and in particular IL-13, in both resting and IL-3 primed basophils. The response was prominent under IL-3 primed condition. However, either SARS-CoV-2 or SARS-CoV-2-infected epithelial cells did not alter the expression of surface markers associated with the activation of basophils, such as CD69, CD13 and/or degranulation marker CD107a. We also validate that human basophils are not permissive to SARS-CoV-2 replication. Though increased expression of immune checkpoint molecule PD-L1 has been reported on the basophils from COVID-19 patients, we observed that SARS-CoV-2 does not induce PD-L1 on the basophils. Our data suggest that basophil cytokine responses to SARS-CoV-2 might help in reducing the inflammation and also to promote antibody responses to the virus.

## Introduction

The current COVID-19 pandemic, caused by the new severe acute respiratory syndrome coronavirus 2 (SARS-CoV-2), presents an unprecedented danger to global health systems, with over 5.2 million confirmed fatalities (10^th^ December 2021) (1). Large number of studies have dissected the spectrum of innate and adaptive immune responses in COVID-19 patients and their role in the immunopathogenesis of the disease (2–7).

Basophils are rare immune cells. In addition to mediating the protection against helminth infection, basophils are well known for their role in the pathogenesis of various allergic inflammatory diseases of respiratory tract, gastro-intestinal tract and skin (8–10). A longitudinal systems-level analyses of immune cells from the blood indicates that basophils are depleted during acute and severe phases of COVID-19 (2) and in line with the established fact that basophils regulate T and B cell responses, a correlation between anti-RBD (receptor-binding domain) IgG titers and basophil number in the circulation has been observed (2). In addition, basophils also displayed an activated phenotype in COVID-19 patients (11). However, the direct response of human basophils to SARS-CoV-2 remains unexplored.

In this study, we have investigated the response of human basophils to SARS-CoV-2 infection. We found that SARS-CoV-2 induces IL-4 and IL-13 cytokines both in resting and IL-3-primed basophils without modifying the expression of surface markers including the checkpoint molecule PD-L1. In fact, PD-L1 expression was at basal level. Our data indicate that activation of basophils by SARS-CoV-2 might support Th2 and antibody responses to the virus.

## Materials and Methods

### Reagents and Antibodies

For flow cytometry, the following fluorochrome-conjugated monoclonal antibodies were used. BD Biosciences (Le Pont de Claix, France): CD13-APC (Clone: WM15), CD69-APC/Cy7 (Clone: FN50), CD274 (PD-L1)-FITC (Clone: MIH1); Miltenyi Biotec: FcϵRIα-PE (Clone: CRA-1); eBioscience (Paris, France): and fixable viability dye eFluor 506. Biolegend (Amsterdam, Netherlands): CD107a-BV421 (clone H4A3). IL-3 was from ImmunoTools (Friesoythe, Germany).

### Cell Lines

Vero E6 (African green monkey kidney epithelial cells, ATCC, CRL-1586) was maintained in Dulbecco’s modified Eagle’s medium (DMEM) containing 10% fetal bovine serum (FBS), 1% penicillin-streptomycin (PS) (5 units/mL penicillin, and 5 μg/mL streptomycin). Caco-2 (Human colon epithelial cells, ATCC, HTB-37) was maintained in DMEM containing 20% FBS, 1% penicillin-streptomycin. All cell lines were cultured at 37°C, 5% CO_2_.

### Virus Strains

The primary strain BetaCoV/France/IDF0372/2020 (EPI_ISL_410720 (GISAID ID); wild-type strain) was supplied by the National Reference Centre for Respiratory Viruses hosted by Institut Pasteur (Paris, France) and headed by Dr. Sylvie van der Werf, and the human sample was isolated and provided by Drs. Xavier Lescure, Yazdan Yazdanpanah from the Bichat Hospital, Paris.

### Virus Production

Viral stocks were produced using Vero E6 in DMEM without FBS with a multiplicity of infection (MOI) 10^-3^ of the virus stock. After 1 h incubation at 37°C under 5% CO_2_, all the medium used for infection was removed before adding fresh DMEM containing 2% FBS, 1% PS. Cells were incubated for further 72 h at 37°C, 5% CO_2_.

### Virus Titration

Viral titers were assessed by plaque assay in 24-well plates on Vero E6 cells (1.5x10^5^ cells/well) in DMEM supplemented with 10% FBS and 1% penicillin-streptomycin. Ten-fold serial dilutions in DMEM without FBS and 1% PS were used for the titration. After 1 h of incubation at 37°C, 5% CO_2_, the medium was replaced by overlaying the cells with carboxymethyl cellulose CMC/DMEM with 2% FBS (vol/vol). After 72 h of incubation, the CMC/DMEM was removed and a mixture of crystal violet/ethanol/formaldehyde was added for 30 min at room temperature. Experiments with live SARS-CoV-2 were performed according to Institut Pasteur guidelines for Biosafety Level 3 work.

### Purification of Basophils

The buffy coats of the healthy donors (Centre Trinité, L’Établissement Français du Sang, Paris; EFS-INSERM ethical committee permission 18/EFS/041) were used to isolate peripheral blood mononuclear cells (PBMCs) by Ficoll density gradient centrifugation. Basophils were isolated from the PBMCs by negative selection using Basophil Isolation Kit II (Miltenyi Biotec, Paris, France).

### Basophil Infection

Freshly isolated basophils were plated in a 96-well plate at a concentration of 0.1x10^6^ cells/100 µl/well without FBS, and directly infected with the SARS-CoV-2 (primary strain IDF0372) at a MOI of one. Non-infected cells were used as a control. In some conditions, basophils were primed with IL-3 (20 ng/ml) along with infection. Vero E6 cells were used as a control of infection. After 1 h, the medium was replaced by fresh X-Vivo medium and the cells were further incubated for 24 h at 37°C, 5% CO_2_. After 24 h of culture, supernatants were collected and stored at -80°C for subsequent cytokine quantification. The cells were processed for surface staining of various markers, such as FcϵR1, CD107a, CD13, CD69, and PD-L1. The cells were fixed with 4% paraformaldehyde and were acquired using LSR II (BD Biosciences). The data were analyzed by BD FACS DIVA and FlowJo software.

### Coculture of Basophils With Virus Infected Caco-2

Human Caco-2 cells were plated a day before in 96-well plates. Cells were infected in DMEM without FBS with the SARS-CoV-2 primary strain IDF0372 at a MOI of one. After 1 h, the medium was replaced by fresh DMEM medium containing 2% FBS, 1% PS and cells were incubated at 37°C, 5% CO_2._ After 24 h, basophils (0.1x10^6^ cells/100 µl/well) were added on the top of infected Caco-2 (5x10^4^ cells/100 µl/well). The basophils were assessed for the expression of various surface markers after 24 h.

### ELISA

Cell culture supernatants were inactivated with Triton X-100 1% (v/v) for 2 h at room temperature. The virus inactivated supernatants were analyzed for the cytokines, such as IL-13 and IL-4 (ELISA Ready-SET-Go, eBioscience).

### Statistical Analysis

As highlighted in the figure legends, the experiments were repeated in several times by using cells from independent donors. Graphs and Statistical analyses were performed by the paired Wilcoxon test (for comparison between two groups) or one-way ANOVA Friedman test with Dunn’s multiple comparisons post-test as indicated using Prism 8 (GraphPad Software Inc, CA). P < 0.05 was considered significant.

## Results

### SARS-CoV-2 Induces Limited Activation of Resting Human Basophils

**Figure 1:**
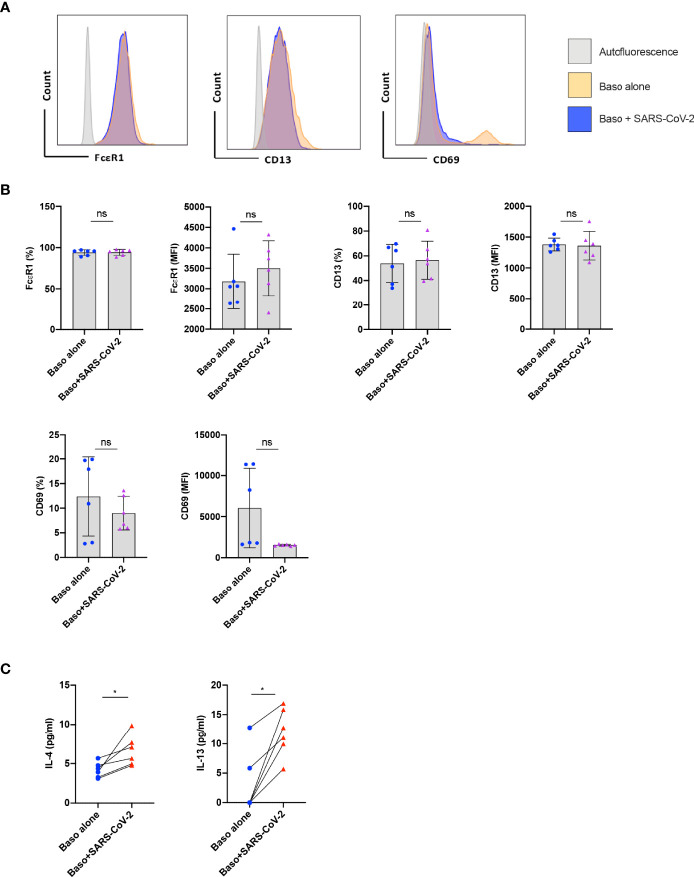
Resting human basophils undergo limited activation upon stimulation with SARS-CoV-2. Basophils (0.1x10^6^ cells/100 µl) isolated from PBMCs of healthy donors were cultured with or without SARS-CoV-2 at a MOI of 1. Cell phenotype was evaluated by flow cytometry after 24 h. **(A)** Representative histogram overlays displaying the expression pattern of FcϵR1, CD13 and CD69 on basophils under various experimental conditions. **(B)** Expression of FcϵR1, CD13 and CD69 on basophils (% positive cells and median fluorescence intensities (MFI), mean ± SD; n = 6 independent donors). **(C)** The amount (pg/ml) of secreted IL-4 and IL-13 in the cell-free supernatant from the above experiments (mean ± SD, n = 6 independent donors). ns, not significant, *P < 0.05, paired Wilcoxon test.

Basophils play a major role in the pathogenesis of various respiratory diseases (8–10). Though the phenotype of basophils was previously analyzed in COVID-19 patients (11), it was not clear whether the activated basophil phenotype observed in the COVID-19 patients was due to direct virus stimulation or a repercussion of inflammatory responses. Therefore, to address this question, we first evaluated the response of basophils to SARS-CoV-2 infection. In our study, we isolated basophils from the healthy donors’ blood and were treated with SARS-CoV-2 for 1 h followed by 24 h incubation. The phenotype of basophils was analyzed by flow cytometry (**Figure 1A**). While the expression of CD13 and CD69 was not modified by SARS-CoV-2, a modest increase in the median fluorescence intensity of FcεRI was observed on SARS-CoV-2-infected basophils (**Figure 1B**). SARS-CoV-2 had no cytopathic effects on the basophils as either cell yield or viability as analyzed by fixable-viable dye were not altered.

We analyzed whether SARS-CoV-2 infection induced cytokine responses in basophils. We found that both IL-4 and IL-13 cytokines were enhanced upon SARS-CoV-2 stimulation (**Figure 1C**) thus confirming SARS-CoV-2 induces activation of human basophils. These data together indicate that resting human basophils undergo limited activation by SARS-CoV-2. Our data also suggest that higher expression of activation markers reported on basophils from COVID-19 patients was possibly not due to the direct virus stimulation (11).

### SARS-CoV-2 Enhances Cytokine Production in IL-3 Primed Basophils

**Figure 2:**
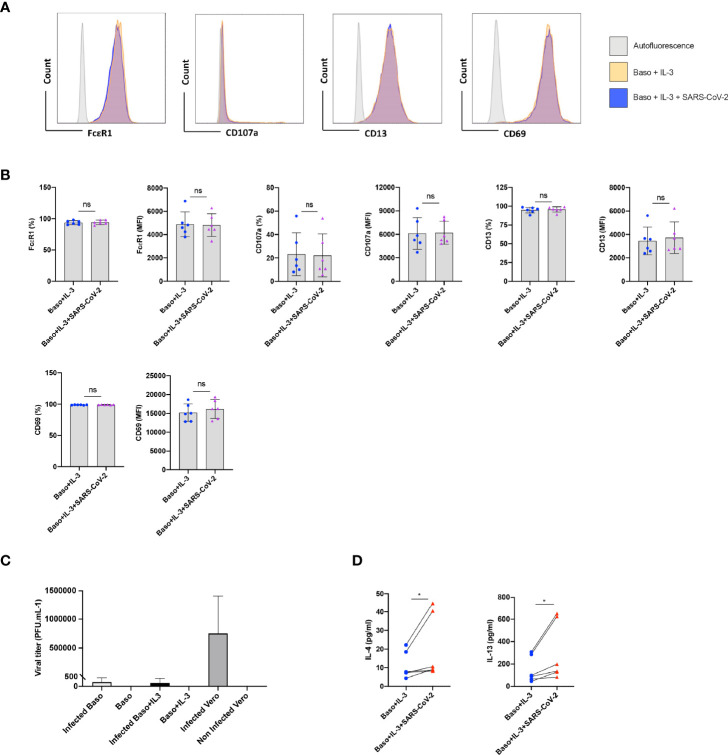
SARS-CoV-2 enhances cytokines in IL-3-primed basophils. Basophils (0.1x10^6^ cells/100 µl) from the healthy donors were primed with IL-3 (20 ng/ml) along with infection with SARS-CoV-2 at a MOI of 1. Non-infected basophils were used as a control. Cell phenotype was evaluated by flow cytometry after 24 h. **(A)** Representative histogram overlays displaying the expression pattern of FcϵR1, CD107a, CD13 and CD69 on basophils under different experimental conditions. **(B)** Expression of FcϵR1, CD107a, CD13 and CD69 on the basophils (% positive cells and median fluorescence intensities (MFI), mean ± SD; n = 6 independent donors). **(C)** Basophils are not permissive to SARS-CoV-2 replication. Either resting basophils (0.1x10^6^ cells/well/96 well plate) or IL-3-primed basophils were infected with SARS-CoV-2 (mean ± SD, n=6 independent donors). Viral titers were evaluated after 24 h post-infection. Infected and non-infected Vero E6 cells were used as controls for the infection (n = 3). **(D)** The amount (pg/ml) of secreted IL-4 and IL-13 in the cell-free supernatant from the above experiments (mean ± SD, n = 6 independent donors). ns, not significant, *P < 0.05, paired Wilcoxon test.

Under certain conditions, basophils require prior priming to undergo activation by stimuli (12). We therefore investigated if SARS-CoV-2 could induce activation of basophils if they were primed. Data from various labs including ours have shown that among various cytokines, IL-3 induces strong priming of human basophils (12–15). Therefore, we primed the basophils with IL-3 along with SARS-CoV-2 stimulation. Priming with IL-3 enhanced the expression of various surface markers (compared to unstimulated basophils, **Figure 1B**) such as CD13 and CD69 (**Figures 2A, B**). However, SARS-CoV-2 did not alter the phenotype of IL-3-primed basophils (**Figure 2B**). Also, SARS-CoV-2 did not induce degranulation of basophils as the expression of CD107a, a marker associated with basophil degranulation, was similar in both the experimental conditions. We confirmed that basophils either resting or IL-3-primed were not permissive to SARS-CoV-2 replication (**Figure 2C**).

Interestingly, SARS-CoV-2 significantly enhanced IL-4 and IL-13 cytokine production in IL-3 primed basophils (**Figure 2D**). Though significant, IL-4 enhancement was marginal compared to IL-13, which is not surprising as IL-4 induction in basophils is dependent on FcεRI-mediated signaling (16).

### Lack of Basophil Activation by SARS-CoV-2 Infected Epithelial Cells

**Figure 3:**
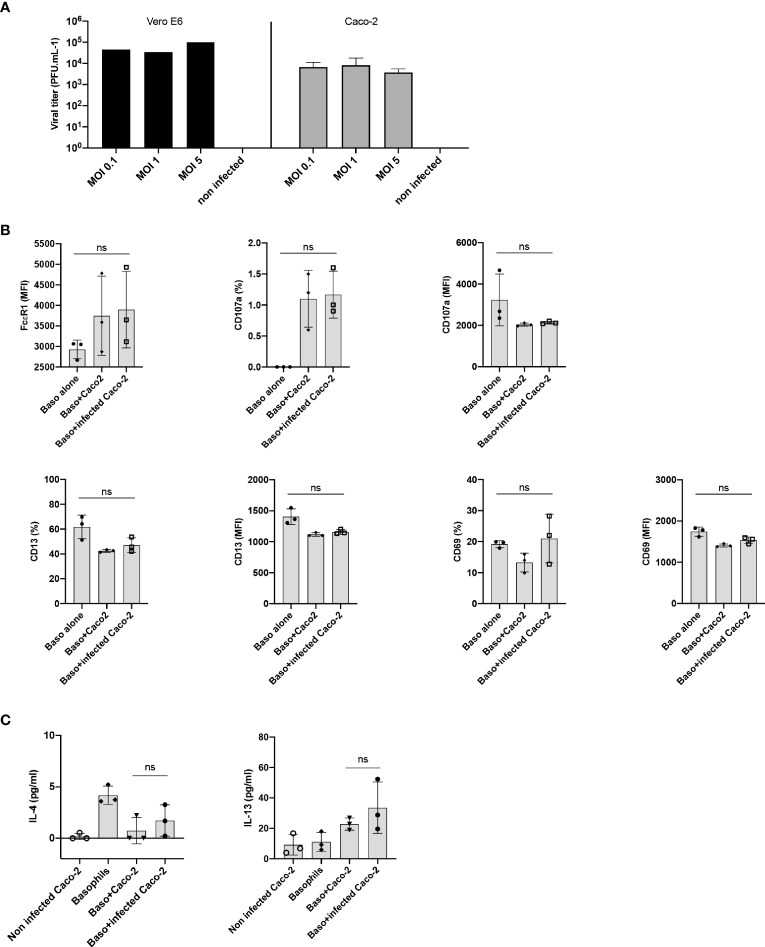
Lack of basophil activation by SARS-CoV-2 infected epithelial cells. **(A)** Caco-2 cells permit SARS-CoV-2 replication. Caco-2 cells (1.5x10^5^ cells per well/24 well plate) in duplicates were infected with SARS-CoV-2 at the MOI of 0.1, 1 and 5. Viral titers were evaluated after 72 h post-infection. Data were presented as mean ± SD. Infection of Vero E6 cells with SARS-CoV-2 was used as a control. **(B, C)** Basophils were cultured as follows: basophils alone (0.1x10^6^ cells/100 µl), basophils (0.1x10^6^ cells/100 µl) + Caco-2 (5x10^4^ cells/100 µl), and basophils + Caco-2 infected with SARS-CoV-2. Cell phenotype was evaluated by flow cytometry after 24 h. **(B)** Expression of FcϵR1, CD107a, CD13, CD69 on basophils (% positive cells and/or median fluorescence intensities (MFI), mean ± SD; n = 3 independent donors). **(C)** The amount (pg/ml) of secreted IL-4 and IL-13 in the cell-free supernatant from the above experiments (mean ± SD, n = 3 independent donors). ns, not significant, one-way ANOVA Friedman test with Dunn’s multiple comparisons post-test.

The entry of SARS-CoV-2 into the cell depends on two receptors, angiotensin-converting enzyme 2 (ACE2) and type II transmembrane serine protease (TMPRSS2), which are involved in binding to RBD of the Spike (S) protein and activation of the S protein by proteolytic priming, respectively. Epithelial cells are the major target of SARS-CoV-2 infection and several studies have highlighted cross-talk between the epithelial cells and basophils (17–19). Therefore, we surmised that epithelial cells infected with SARS-CoV-2 could affect activation of basophils. Though A549 lung epithelial cells have been reported to induce activation of human basophils (18), they lack both ACE2 and TMPRSS2 receptors (20). On the other hand, human colorectal adenocarcinoma cells (Caco-2 cells) were reported to express both ACE2 and TMPRSS2 receptors and support SARS-CoV-2 replication (20). Virus titration experiments have confirmed that Caco-2 are permissive to SARS-CoV-2 infection (**Figure 3A**). However, SARS-CoV-2-infected Caco-2 cells did not alter either phenotype of basophils (**Figure 3B**) or cytokines secreted (**Figure 3C**).

### SARS-CoV-2 Does Not Induce PD-L1 on the Basophils

**Figure 4:**
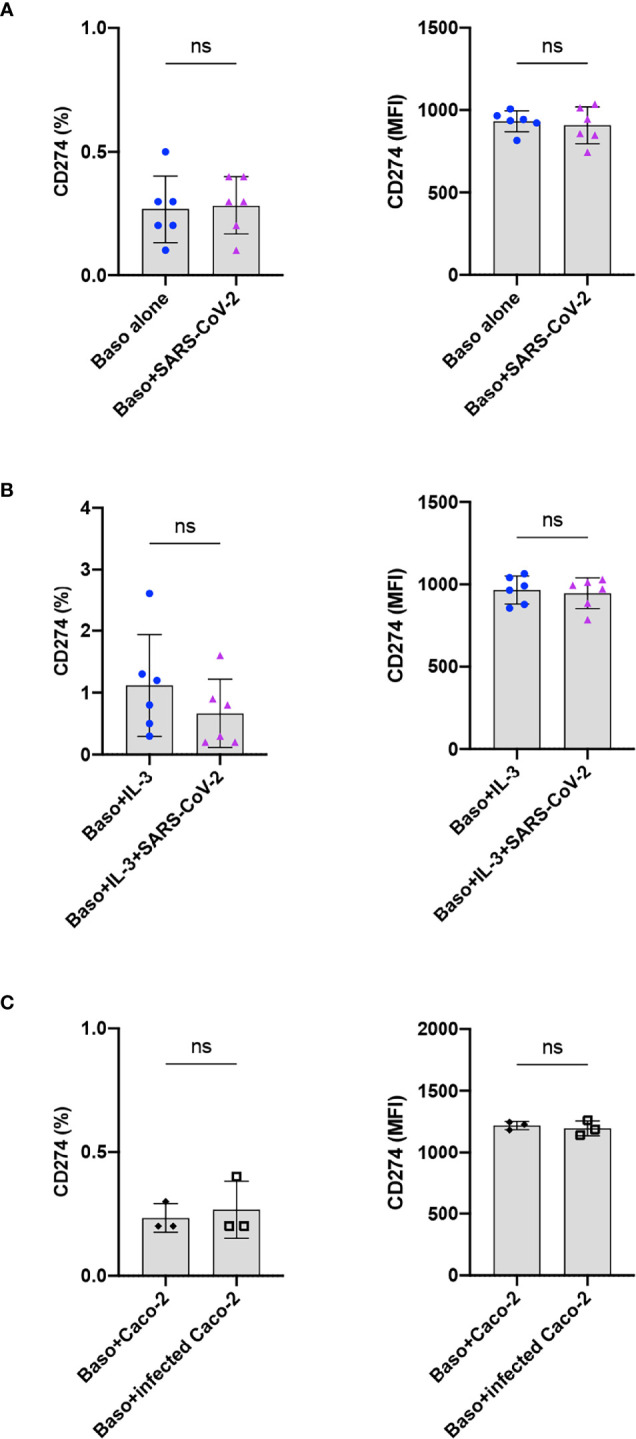
SARS-CoV-2 does not induce PD-L1 on the basophils. The expression of PD-L1 on the basophils under various experimental conditions of SARS-CoV-2 stimulation. **(A)** Resting basophils (0.1x10^6^ cells/100 µl) were cultured with or without SARS-CoV-2 for 24 h and the expression of PD-L1 was analyzed by flow cytometry. **(B)** Basophils were primed with IL-3 along with infection with SARS-CoV-2. Non-infected basophils were used as a control. PD-L1 was analyzed after 24 h culture. **(C)** Basophils were cultured with either non-infected or SARS-CoV-2 infected Caco-2 cells for 24 h. The data (mean ± SD) were presented as % cells positive for PD-L1 and median fluorescence intensities (MFI), and were from 3 **(C)** to 6 **(A, B)** independent donors. ns, not significant, paired Wilcoxon test.

Immuno-phenotyping studies have revealed that programmed death receptor ligand 1 (PD-L1, CD274, B7-H1; family of B7 costimulatory molecule) is dysregulated on monocytes, neutrophils, and T cells in COVID-19 patients (21). In addition, an enhanced expression of PD-L1 was also observed on the peripheral blood basophils of severe COVID-19 patients (11, 22–24), though this observation was not confirmed by another report (11). Therefore, we investigated the repercussion of SARS-CoV-2 infection on the expression of PD-L1 on the basophils at 24 h. We found that both resting as well as SARS-CoV-2-infected basophils lacked the expression of PD-L1 (**Figure 4A**). Similar results were also obtained with IL-3-primed basophils (**Figure 4B**) and basophils co-cultured with virus-infected Caco-2 (**Figure 4C**). Our control experiments have indicated that the expression of PD-L1 on the basophils did not differ at 24 and 48 h. These data together suggest that SARS-CoV-2 does not induce PD-L1 on the basophils and altered expression of this molecule observed on the basophils from COVID-19 patients was possibly due to the inflammatory cytokine responses.

## Discussion

Various studies have confirmed the dysregulation of immune system, particularly cells of the innate immune system and cytokine storm in severe COVID-19 patients (25). Systems-level immuno-monitoring of adult COVID-19 patients by mass-cytometry revealed that polymorphonuclear granulocytes (neutrophils, eosinophils, and basophils) are differentially regulated in the circulation during infection (2). Both absolute count and relative frequencies of basophils are decreased in moderate and severe COVID-19 patients (11, 26). However, in contrast to neutrophils, basophils are increased from acute to recovery phase though another cohort reported no difference in the basophil count in COVID-19 patients during acute phase as compared to healthy donors (27). An activated phenotype of basophils was also documented in COVID-19 patients (11) though the underlying mechanisms are not known. In view of activation of complement pathway in COVID-19 patients (28–30) and that human basophils express C5aR and C3aR (31), indirect activation of basophils in COVID-19 patients through complement components cannot be ruled out. However, direct or indirect interaction of basophils with SARS-CoV-2 has not been investigated yet, despite their vital role in the pulmonary pathologies and regulation of immune responses. The low frequency of basophils (0.2 to 0.5% of leukocytes) in the circulation is one of the main limitations for researchers to study the basophils.

Direct capture of human immunodeficiency virus (HIV)-1 by basophils has been described (32), though the identity of pattern recognition receptor (PRR) that mediates virus capture is not clear (33). Human basophils produce histamine when cells are incubated with paramyxoviruses, thus indicating that basophils have a capacity to respond to virus stimuli (34). In addition, human basophilic cell line (KU812) has been demonstrated to be permissive to rhinovirus infection *in vitro* (35). In view of these observations and the role of basophils in various respiratory pathologies, we investigated the cross-talk between SARS-CoV-2 and basophils. Our data suggest that SARS-CoV-2 could induce partial activation of basophils leading to the secretion of cytokines, IL-4 and IL-13, both in resting and IL-3-primed basophils. The effect of SARS-CoV-2 on IL-13 induction was particularly remarkable in IL-3 primed condition. The mechanism by which SARS-CoV-2 induces basophil activation is the subject of future investigation.

Previous data have shown inability of SARS-CoV-2 to replicate in various immune cells (36). Also, proteomic and genomic data clearly highlighted the absence of ACE2 receptor on the basophils (37). Therefore, we believe that SARS-CoV-2 could induce basophil activation by signaling through PRR in coordination with cytokines like IL-3. Activated T cells are the major source of IL-3 (14, 38, 39) and flow cytometric data showed that IL-3 in COVID-19 patients is contributed mainly by CD4^+^ T cells (40). Of note, low levels of IL-3 in the circulation of COVID-19 patients is associated with enhanced severity of the disease and mortality (40). Mechanistically, it was proposed that IL-3 enhances the recruitment of plasmacytoid dendritic cells into the airways and hence boosts the anti-viral innate immunity. Our data imply that IL-3 also primes basophils to secrete higher amounts of cytokines IL-13 and IL-4 in response to virus stimulation that might help in reducing the inflammation and also to promote antibody responses to the SARS-CoV-2.

Interaction between the checkpoint molecules programmed cell death protein PD-1 and PD-L1 plays a key role in maintaining immune tolerance (41–43). However, immune exhaustion by signaling through checkpoint molecules could prevent effective clearance of the pathogens leading to exacerbated immune responses (44, 45). PD-L1 and PD-1 interaction also supports regulatory T cell responses (46–50). Of note, PD-L1 expression was significantly higher in a group of severe COVID-19 patients as compared to milder patients and was positively correlated with the WHO and Sequential Organ Failure Assessment (SOFA) clinical scores (22). It is likely that the induced expression of PD-L1 in severe COVID-19 patients might be responsible for the T cell exhaustion. The trigger that induces PD-L1 on the basophils from COVID-19 patients is not known. Our data however suggest that SARS-CoV-2 infection does not induce PD-L1 on the basophils (51). However, PD-L1 on basophils may not influence the effector CD4^+^ T cell responses as basophils lack the features of antigen presenting cells (52–56). In view of these facts, the significance of basophil-PD-L1 in the pathogenesis of COVID-19 remains unclear.

SARS-CoV-2 entry is facilitated by the presence of ACE2 and TMPRSS2 receptors on the host cells. These receptors are highly expressed by epithelial cells that are present in the lungs and gastrointestinal tract. Several innate stimuli including epithelial derived inflammatory cytokines (IL-33, IL-18, Thymic stromal lymphopoietin, and GM-CSF), growth factors (IL-3, IL-7, TGF-β, and VEGF) activate the mouse basophils (57). Also, airway epithelial cell line A549 has been reported to induce activation of human basophils (18). Whether other epithelial cells also display similar capacity to induce basophil activation is not known. Our results however suggest that Caco-2 cells lack the ability to induce basophil activation. Primary lung epithelial cells need to be used to examine the cross-talk between SARS-CoV-2-infected airway epithelial cells and basophils, and is the limitation of our study.

## Data Availability Statement

The original contributions presented in the study are included in the article/supplementary material. Further inquiries can be directed to the corresponding author.

## Ethics Statement 

EFS-INSERM permission (18/EFS/041) was obtained for the purchase of healthy donors buffy coats used in the study.

## Author Contributions

JB conceptualized, designed and coordinated the study, contributed to the interpretation of the results and wrote the paper. SB, CC, LL, and MJM performed the experiments and participated in the data interpretation and wrote the first draft of the paper. AS coordinated the study along with JB. All authors read and approved the final submitted version of the article.

## Funding

Agence Nationale de la Recherche, France under the call “Flash COVID-19” (ANR-20-COVI-0093-COVIMUNE) and ANR-19-CE17-0021 (BASIN).

## Conflict of Interest

The authors declare that the research was conducted in the absence of any commercial or financial relationships that could be construed as a potential conflict of interest.

## Publisher’s Note

All claims expressed in this article are solely those of the authors and do not necessarily represent those of their affiliated organizations, or those of the publisher, the editors and the reviewers. Any product that may be evaluated in this article, or claim that may be made by its manufacturer, is not guaranteed or endorsed by the publisher.
